# Sex Is Not an Independent Predictor of Exercise-Induced Pain After Adjustment for Performance and Pain Sensitivity

**DOI:** 10.1155/prm/6147802

**Published:** 2025-11-17

**Authors:** Suzana Bojic, Nemanja Radovanovic, Milica Radovic, Maja Stojanovic, Marija Stevic, Marko Djuric, Nemanja Dimic, Dusica Stamenkovic

**Affiliations:** ^1^Department of Anesthesiology and Intensive Care, Faculty of Medicine, University of Belgrade, Belgrade, Serbia; ^2^Department of Anesthesiology and Intensive Care, University Clinical Center of Serbia, Belgrade, Serbia; ^3^Department of Internal Medicine, UCHC Zemun, Belgrade, Serbia; ^4^University of Defence, Medical Faculty of the Military Medical Academy, Belgrade, Serbia

**Keywords:** athletes, cold pressor test, exercise-induced pain, pain threshold, pain tolerance, sex

## Abstract

**Introduction:**

Exercise-induced pain (EIP) is a transient pain phenomenon that emerges during physical exertion and resolves soon after exercise cessation. Despite being recognized as a performance-limiting variable in endurance sports, the mechanisms driving its interindividual variability remain poorly defined. We aimed to determine whether sex remains a significant predictor of EIP intensity after adjustment for performance and pain sensitivity.

**Materials and Methods:**

This cross-sectional study enrolled 122 recreational athletes (61 males and 61 females), including 48 trail runners and 74 hikers. Participants provided self-reported data on demographics and training habits. Performance was evaluated using both external and internal load metrics: external load was represented by activity duration and effort-adjusted speed, while internal load was assessed using the rating of perceived exertion (RPE). Maximum and average EIP was rated on a numeric rating scale and combined into a pain composite score (PCS). Pain threshold and tolerance were measured using the cold pressor test. Associations between variables and pain outcomes were analyzed using generalized linear models.

**Results:**

No significant differences were observed in maximum or average EIP intensity, pain threshold, or pain tolerance between male and female athletes. Sex was not a significant predictor of the PCS after adjusting for external and internal exercise load. Neither pain threshold nor tolerance significantly predicted PCS, and these associations did not vary by gender.

**Conclusion:**

In recreational endurance athletes, sex had no significant impact on EIP intensity when accounting for performance and pain sensitivity. These findings challenged traditional assumptions about sex-related pain differences.

## 1. Introduction

Exercise-induced pain (EIP) is a distinct, transient pain phenomenon that emerges during physical exertion and typically resolves soon after exercise cessation [1]. Unlike injury-related pain and delayed onset muscle soreness (DOMS), which involve tissue damage and inflammation, EIP is driven by the accumulation of noxious metabolites in the working muscles, without underlying tissue injury [1]. Despite being recognized as a performance-limiting variable in endurance sports [2], the mechanisms driving interindividual variability in EIP remain poorly defined.

EIP intensity reliably scales with objective exercise load, particularly power output [3] and activity duration [4]. However, most supporting data come from controlled laboratory studies, limiting their ecological validity, especially in noncycling endurance sports and real-world performance contexts. Notably, perceived pain does not always match objective workload [5], indicating that EIP is influenced by factors beyond physical effort. Among these, sex remains insufficiently examined despite its established relevance in broader pain research. While females generally report greater pain sensitivity and lower tolerance [6], it remains unclear whether these differences persist in the context of EIP [7], and, if so, whether they stem from biological factors associated with sex [8], sociocultural factors associated with gender [9], or both. The binary structure of sport, along with ongoing debates over transgender athlete inclusion, further complicates efforts to disentangle sex and gender in exercise and pain research. Similarly, baseline pain threshold and tolerance are assumed to shape pain during exertion, yet their predictive value for EIP is uncertain. While endurance athletes consistently exhibit higher thresholds and tolerance compared to nonathletes [10], these findings largely reflect elite populations with specialized physiological (e.g., aerobic capacity and muscle adaptations) and psychological adaptations (e.g., resilience and stress regulation) [11–13]. In contrast, recreational endurance athletes, despite their rapidly growing numbers and closer resemblance to the general population, remain underrepresented in this field, even though they provide a valuable context for exploring how sex and pain sensitivity influence the experience of EIP.

We hypothesized that biological sex is not an independent determinant of EIP intensity in recreational endurance athletes and that apparent sex differences are confounded by individual differences in performance and baseline pain sensitivity. This study aimed to determine whether sex remains a significant predictor of EIP intensity after adjustment for these confounders and to quantify their relative contributions to EIP variability.

## 2. Materials and Methods

### 2.1. Participants and Recruitment

This cross-sectional, observational study was approved by the Institutional Ethics Board (Decision No. 01-49/1-2022, dated May 4, 2022). All participants provided written informed consent. Data collection occurred between June 1, 2022, and June 1, 2024. Field researchers approached participants at organized trail running and hiking events held in two neighboring countries, Republic of Serbia and Republic of Montenegro, with the approval of event organizers.

To ensure a controlled assessment of acute pain responses, we applied the following exclusion criteria for sport events: (i) events occurring above 2500 m above sea level, to avoid the confounding effects of hypoxia on pain perception; (ii) multiday events, to eliminate variability caused by cumulative fatigue, sleep deprivation, and prolonged stress; (iii) winter weather conditions, to control for environmental factors; and (iv) trails with any section rated above T2 on the Swiss Alpine Club (SAC) Mountain and Alpine Hiking Scale. Trails exceeding T2 rating involve pathless sections, significant exposure, steep terrain, or require the use of technical equipment such as ice axes, ropes, or crampons [14].

Inclusion criteria for participants were (i) age ≥ 18 years, (ii) native Serbian/Montenegrin speakers, (iii) participation in trail running or hiking events in the above-mentioned countries, and (iv) provision of signed informed consent. Participants' exclusion criteria were (i) preexisting acute or chronic pain conditions and (ii) professional athlete status at any point in life.

### 2.2. Demographic Data

Demographic variables included chronological age (years), standing height (cm), and body mass (kg), all obtained via structured self-report questionnaires. Body mass index (BMI) was calculated using the standard formula: BMI = body mass (kg)/[height (m)]^2^.

Self-reported sex assigned at birth (male/female) was recorded. We did not assess gender identity due to the linguistic constraints of Serbian/Montenegrin, where “pol” (пол) lacks lexical differentiation between biological sex and sociocultural gender. While we report findings by sex in accordance with Sex and Gender Equity Research (SAGER) guidelines, we acknowledge the conceptual overlap and recognize gender identity's significant role in shaping pain experiences [15].

### 2.3. Training Data

Training metrics were evaluated through self-reported questionnaires that gathered information on weekly training hours, the number of training days per week, and the total years of participation in any sport.

### 2.4. Performance Metrics

Performance metrics included measures of external (objective) and internal (subjective) exercise load.

To assess external load, field researchers recorded trail distance (km), cumulative elevation gain (m), and completion time for each participant. Effort-adjusted speed (km-effort/h), a key external load metric, was then calculated as follows: [(trail distance + (elevation gain (m)/100)]/time (h) [16]. Activity duration served as an additional external load indicator.

As a measure of internal load, participants rated perceived exertion immediately postactivity using an 11-point rating of perceived exertion (RPE) scale (0 = “rest”; 10 = “maximal exertion”) [17].

### 2.5. EIP Assessment

After completing the sporting event, athletes were asked to rate their maximum and average EIP pain intensity during the activity using a 11-point numeric rating scale (NRS) (0 indicated “no pain” and 10 represented “the worst pain imaginable”). These two scores were then used to compute the pain composite score (PCS), which was developed to capture the overall pain experience by integrating both peak and sustained pain intensity [18]. Specifically, PCS was calculated as the arithmetic mean of the maximum and average EIP, i.e., PCS = (maximum EIP + average EIP)/2.

### 2.6. Pain Threshold and Tolerance

Pain threshold and tolerance were assessed using the cold pressor test (CPT) [19]. Participants immersed their nondominant hand into a container of circulating cold water maintained at a constant temperature of 1°C–4°C. They were instructed to keep their hand submerged until they either reached their pain tolerance limit or 180 s had elapsed. Pain threshold was recorded as the time from immersion to the first perception of pain, while pain tolerance was defined as the total duration the hand remained in the water. In addition, pain intensity was evaluated at 20 s intervals throughout the test using NRS [20]. These repeated measurements allowed for a detailed temporal profile of pain intensity during the test. The PCT was conducted between 6:00 and 10:00 a.m., prior to the start of the sporting event to ensure a baseline measurement unaffected by physical exertion or circadian rhythm. Pain threshold and tolerance values were measured only in trail runners due to technical limitations.

### 2.7. Statistical Analysis

Data analysis was conducted using IBM SPSS Statistics for Windows, Version 22.0 (IBM Corp., Armonk, NY, USA). The Kolmogorov–Smirnov test was used to assess the normality of data distributions. Continuous variables were reported as medians with interquartile ranges (25^th^−75^th^ percentiles), and group differences were analyzed with the Mann–Whitney *U* test. Categorical variables were expressed as frequencies, and group differences were evaluated using the Chi-square test. Generalized linear models with a gamma distribution and log link function were employed to identify predictors of the PCS, which integrates maximum and average EIP intensity. This approach was selected to account for the skewed nature of the pain intensity data, which included non-negative values with a right-skewed distribution. To address the presence of zero values and prevent computational issues, a small constant (0.01) was added to all PCS scores [21]. A *p* value of less than 0.05 was considered statistically significant.

## 3. Results

The data presented in Table 1 reveal statistically significant differences between trail runners and hikers, particularly in sex distribution and performance measures. A higher proportion of male participants was observed among trail runners, although the groups did not differ significantly in age or BMI. Trail runners exhibited markedly greater performance and training metrics, reflecting higher external load (effort-adjusted speed) and internal load (RPE), alongside significantly greater training volume and frequency. Critically, trail runners reported significantly higher EIP intensity than hikers, as evidenced by elevated maximum and average pain ratings and, consecutively, higher PCS.

As summarized in Table 2, male and female athletes were comparable in age, although males had a higher BMI. Male athletes also reported greater weekly training load and demonstrated higher effort-adjusted speed; however, RPE did not differ between sexes. Notably, PCS values were similar across groups, with no statistically significant sex-based differences observed.

**Figure 1:**
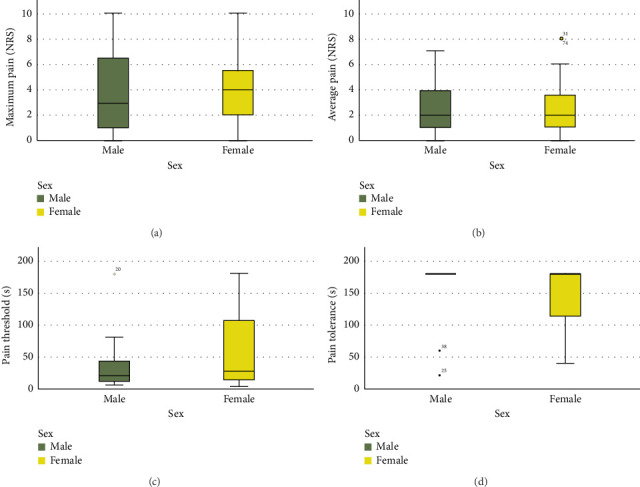
Sex differences in (a) maximum exercise-induced pain, (b) average exercise-induced pain, and (c) pain threshold and (d) pain tolerance in recreational endurance athletes. *Note:* Error bars represent interquartile ranges, with median values indicated by horizontal lines within each box. None of the comparisons between sexes yielded statistically significant differences (Mann–Whitney *U* tests, *p* > 0.05).

As presented in Figure 1, no statistically significant differences were observed between sexes across pain metrics (*p* > 0.05), including maximum and average EIP intensity, pain threshold, and pain tolerance in male and female recreational endurance athletes.

Univariate predictors of EIP intensity, operationalized via the PCS, are presented in Table 3. Female sex, older age, and higher BMI were each associated with higher PCS values, indicating greater reported pain. Training load, including weekly training hours, training frequency, and years of experience, also showed positive associations with PCS, as did performance metrics such as effort-adjusted speed, activity duration, and RPE. Among these, RPE exhibited one of the strongest associations with PCS, indicating that each one-point increase in perceived exertion was associated with a 22% increase in reported pain intensity. In contrast, pain threshold and pain tolerance were not significant predictors of PCS.

Table 4 presents multiple regression models using the PCS, which integrates maximum and average EIP intensity, as the dependent variable. In all models, sex was not a significant predictor of EIP intensity when adjusted for sport type, external load (effort-adjusted speed or activity duration), and internal load (subjective exertion). Instead, higher perceived exertion was consistently associated with greater EIP intensity, with each one-point increase in RPE corresponding to an estimated 23% increase in PCS. In contrast, external load measures such as effort-adjusted speed and activity duration had negligible effects on PCS.

We also conducted regression analyses that included sex along with each individual predictor of EIP intensity (age, BMI, hours of training per week, days of training per week, and years of training). In all models, sex was not a significant predictor of EIP intensity when adjusted for each demographic or training-related parameter individually.

**Figure 2:**
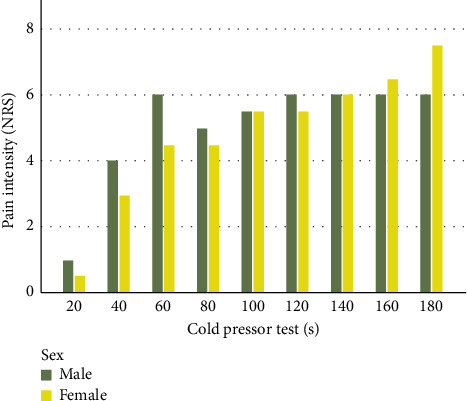
Temporal dynamics of pain intensity during cold pressor test. *Note:* The height of each bar indicates the median pain intensity values at each time point of the cold pressor test.

The Mann–Whitney *U* test revealed no statistically significant differences between male and female athletes at any time point of CPT, with *p* values exceeding 0.05 for all comparisons (Figure 2).

Our results indicate that neither pain threshold nor pain tolerance significantly predicts EIP intensity, and these relationships do not vary by sex (Table 5). Sex was not a significant predictor in these models, and no interaction effects between sex and pain metrics were observed. These findings suggest that EIP intensity is independent of pain threshold, pain tolerance, and their interactions with sex.

## 4. Discussion

The central finding of this study is that sex was not a significant predictor of EIP intensity after adjusting for performance metrics and pain sensitivity. Additionally, no sex differences were observed in pain threshold or tolerance. While sex differences in pain are often assumed, particularly in athletic contexts, the potential confounding effects of performance and pain sensitivity have not been systematically addressed until now. By accounting for these factors, this study offers a more nuanced perspective on the relationship between sex and EIP, contributing novel insight to an underexplored area.

While men and women differ in their perception and processing of musculoskeletal pain, these differences are often context-specific and not uniformly expressed across populations or pain modalities. Clinically, women report a higher prevalence of musculoskeletal pain disorders, such as fibromyalgia, myofascial pain, and complex regional pain syndrome, and tend to experience greater functional impairment [22]. Females also exhibit lower pain thresholds and tolerances, alongside heightened sensitivity to mechanical and thermal stimuli [23]. These differences arise from distinct physiological mechanisms, including sex-specific roles of genes, proteins, hormones, and immune system interactions that modulate pain transmission [24]. Neuroimaging further highlights sex-specific brain alterations in both chronic [25] and acute pain conditions [26]. Surprisingly, studies focusing on sex or gender differences in pain perception specifically among athletes remain scarce and inconclusive [27]. In one of the few available studies, Dannecker and colleagues assessed both EIP and DOMS following bouts of eccentric muscle contractions. They reported lower pain intensity in women compared to men, though sex differences in muscle damage biomarkers did not reach significance [7]. However, this research was conducted under controlled laboratory conditions using strength-based exercises, which may limit ecological validity for endurance athletes.

Traditionally, females are reported to have lower pain threshold and tolerance than males [6, 28]; however, our findings challenge this notion, as we observed no sex differences in responses to CPT, a model of sustained cold-induced pain that activates peripheral nociceptors and engages central pain modulatory pathways. Similarly, Kowalczuk et al. found no consistent sex differences in cold pain threshold or tolerance, though normally menstruating women showed greater adaptation over repeated exposures, suggesting a role for gonadal hormones in pain modulation [29]. In contrast, Diotaiuti et al. reported that female athletes had higher initial pain ratings during the CPT; however, overall pain perception was more strongly influenced by the interaction between sex and competitive experience, rather than by sex alone [30], highlighting the nuanced and context-dependent nature of sex differences in pain perception.

Trail runners in our study demonstrated exceptionally high pain tolerance, with nearly all participants enduring the CPT to its maximum duration, closely mirroring the findings reported by Pettersen et al. [31]. This aligns with findings from a recent systematic review and meta-analysis by Thornton et al., which demonstrated that athletes, particularly those in endurance disciplines, consistently show greater pain tolerance and report lower pain intensity during experimentally induced pain, including cold-based protocols [10]. Such resilience may reflect the unique physiological and psychological demands of endurance training, where pain is not conceptualized as a threat, but as a marker of effort and success [32], fostering a more adaptive response to noxious input. Additionally, exposure to variable environmental stressors, such as fluctuating temperatures and terrain, may contribute to conditioning effects that enhance cold pain tolerance. Given evidence that pain sensitivity varies with stimulus modality [33], future work should investigate whether these effects generalize across thermal and mechanical stimuli in ecologically valid, real-world contexts.

Our study did not account for hormonal status in female participants—a decision that may appear controversial but reflects a deliberate and pragmatic choice. While we fully acknowledge the substantial influence of hormonal fluctuations (e.g., menstrual cycle, menopause) on pain perception [6], we also recognize that female athletes routinely train and compete across all cycle phases. In this context, controlling for hormonal status may offer limited practical insight and risks pathologizing normal biological variability. Future studies might address this issue with more nuance, but we argue that performance-based research in athletes should prioritize real-world applicability over laboratory precision when hormonal effects are unlikely to alter behaviorally relevant outcomes.

We hypothesized that both objective measures of exercise load (effort-adjusted speed and activity duration) and subjective load (RPE) would predict the intensity of EIP. While the association between external load and EIP is well documented [1], RPE emerged as a markedly stronger predictor in our analysis. Although conceptually distinct, EIP and RPE frequently converge during prolonged or high-intensity exercise, likely reflecting overlapping perceptual and neurophysiological mechanisms [34]. Prior work by Hollander et al. demonstrated parallel increases in RPE and EIP with increasing workload, though such patterns do not fully explain interindividual variability [35]. Psychological factors, such as anxiety, depression, and personality traits, are increasingly recognized as modulators of perceived exertion, shaping how individuals interpret and report internal sensations [33–38]. It is plausible that similar mechanisms influence the relationship between RPE and EIP, warranting further investigation in future research.

A significant gap in the current understanding of pain in athletic population lies in the overrepresentation of elite and professional athletes in the literature. Unique physiological (e.g., aerobic capacity, aerobic power, muscle-specific adaptations) and psychological adaptations (e.g., resilience, stress management) of elite athletes [11–13], while advantageous for performance, restrict the applicability of findings to broader populations. Functional imaging studies have demonstrated that these adaptations are associated with enhanced pain processing in brain regions such as the anterior cingulate cortex [32, 39]. This is further supported by evidence showing that elite endurance athletes exhibit significantly higher pain thresholds and tolerances compared to nonathletes and athletes in other disciplines [31, 40, 41]. In contrast, recreational athletes, who lack elite-specific adaptations, are underrepresented in pain research, despite being far more numerous and driving the recent surge in endurance sports participation [42]. Including this population is essential for a more inclusive and ecologically valid understanding of EIP, especially regarding sex- and gender-related differences.

Trail runners and hikers were intentionally sampled to capture a large variability of performance metrics we deemed relevant to the development of EIP. This strategy was informed by evidence demonstrating that, even at elite level, these disciplines exist on a physiological and biomechanical continuum as trail runners frequently hike steep ascents [43]. Although distinct in absolute performance measures (e.g., effort-adjusted speed and RPE), their shared physiological demands provide a robust natural model for examining performance-related influences on EIP. While advanced metrics such as VO_2_ max, lactate threshold, or running economy provide a more nuanced understanding of exercise load, these are typically unavailable in recreational athletes and were not part of our dataset. This highlights the need for future studies to integrate such advanced metrics to better understand performance and pain experiences in nonelite populations.

No upper age limit was set a priori, as the study aimed to reflect the real-world demographic profile of recreational endurance athletes in Serbia and Montenegro. Based on prior experience and community involvement, individuals over the age of 60 were not expected to meet inclusion criteria, which was confirmed by the absence of such participants in the final sample.

The wide range observed in pain-related boxplots reflects substantial interindividual variability, which is expected in real-world field settings where physiological, psychological, and environmental factors interact [1]. Unlike controlled lab environments, outdoor endurance events introduce natural fluctuations that enhance ecological validity. Statistically, this heterogeneity was addressed using generalized linear models with a gamma distribution and log link, which handle right-skewed, non-normal data effectively. These models allow for accurate estimation of effects despite large dispersion in outcome variables [44].

### 4.1. Limitations

In this study, only sex assigned at birth was recorded via self-report, while gender identity was not assessed. This reflects a linguistic constraint, as Serbian and Montenegrin use a single term “pol” (пол) for both sex and gender, with no validated tools to distinguish them. While this limits our ability to capture the influence of gender identity, an important factor in pain perception, it ensured cultural and methodological consistency. Future research should prioritize the development of locally adapted, gender-sensitive measures to address this gap.

Our sample consisted of recreational endurance athletes from Serbia and Montenegro, two neighboring countries with a shared language and highly similar sociocultural and historical backgrounds. While this setting offers a cohesive context for studying pain and exertion in real-world endurance events, the use of a convenience sample introduces potential bias. Participants were not randomly selected, which may result in overrepresentation of certain demographic or psychological profiles and limit the generalizability of our findings to broader populations.

Although we adjusted for training volume using self-reported weekly hours, this metric did not capture training intensity, terrain, or objective external load indicators (e.g., GPS-based data and power output), which could affect pain and exertion. Although weekly training hours were collected as a general indicator of training volume, this measure alone does not reflect the full complexity of external training load. In trail running, relevant external load variables include distance covered, elevation gain, pace, power output, and GPS-derived metrics such as speed variability or terrain characteristics. Additionally, accelerometer-based data (e.g., stride dynamics, acceleration/deceleration patterns) can provide further insight into mechanical demands. However, such detailed data were not consistently available in our sample, as recreational athletes often lack access to standardized monitoring tools or do not track their training systematically. This limitation is consistent with prior observations by Bourdon et al., who noted that training load monitoring in nonelite populations remains constrained by the absence of valid, standardized, and field-applicable measurement tools [45]. Moreover, beyond its impact on physiological performance, structured training, particularly prolonged or high-intensity sessions, may enhance an athlete's tolerance to exercise-induced discomfort and pain, through mechanisms involving habituation, altered pain processing, and psychological adaptation [32, 46, 47]. As such, while training hours appeared similar between groups, unmeasured differences in training intensity, terrain, and modality could have influenced both pain perception and performance outcomes.

Additionally, body composition was estimated via BMI rather than more precise measures such as body fat percentage or lean mass. The minimal differences in BMI suggest that this metric alone is insufficient to explain performance differences, as a more precise measure of body composition, such as body fat percentage or lean muscle mass, would provide a better understanding of its impact [48].

Although we collected data on potential modulators of pain perception, such as caffeine and analgesic use, these variables could not be incorporated into the analysis due to limitations in data quality. Participants were asked to report any caffeine intake (e.g., coffee, energy drinks, and caffeinated gels) and analgesic use within a pharmacologically relevant time frame on the day of the event. While caffeine consumption was common, primarily in the form of morning coffee and, among trail runners, caffeine-containing sports supplements, participants often failed to provide sufficient detail to calculate dosage. Similarly, analgesic use was infrequent. Despite our efforts to capture this information, the variability and incompleteness of responses precluded meaningful statistical evaluation. As such, we acknowledge the inability to account for these factors as a limitation of the study.

Lastly, the cross-sectional nature of our study inherently limits the ability to draw causal inferences regarding the observed associations between EIP and other observed variables. Additionally, our assessment focused solely on cold-induced pain, without evaluating other clinically relevant pain modalities such as heat, pressure, or ischemia [34]. Important dimensions of pain experience, including unpleasantness, affective distress, and temporal summation, were also not captured [34]. Moreover, the CPT was administered only to trail runners due to logistical constraints at event sites, which restricts the generalizability of our findings and precludes direct comparisons between trail runners and hikers. Another notable limitation is the absence of exercise-induced hypoalgesia (EIH) assessment, which may have influenced pain responses differentially depending on activity type, fitness level, or psychological traits [49, 50]. Future studies should incorporate multimodal pain testing, longitudinal designs, and EIH assessment to more comprehensively characterize the dynamic relationship between exercise, exertion, and pain perception.

## 5. Conclusions

After adjusting for performance metrics and baseline pain sensitivity, sex was not a significant predictor of EIP intensity, nor were sex differences observed in cold pain threshold or tolerance. These results challenge common assumptions about sex differences in athletic pain perception and highlight the importance of contextual and individual factors. Future research should prioritize longitudinal designs and include diverse athletic populations beyond the elite level, while incorporating hormonal profiling, multimodal pain assessments (e.g., heat, pressure, and ischemia), and measures of EIP. Capturing broader pain dimensions, such as unpleasantness and affective distress, will further clarify how individual, physiological, and contextual factors interact to shape pain experiences during exercise.

## Figures and Tables

**Table 1:** Comparison of demographics, training habits, and performance and pain metrics between trail runners and hikers.

	Trail runners (*N* = 48)	Hikers (*N* = 74)	*p* value
Demographics			
Sex (male/female)	29/19	32/42	0.047
Age (years)	39.00 [35.00–45.50]	40.00 [35.00–47.00]	0.386
BMI (kg/m^2^)	22.74 [20.78–23.87]	22.60 [20.81–25.14]	0.304
Training			
Hours per week	8.00 [5.00–10.00]	4.00 [2.00–7.00]	< 0.001
Days per week	5.00 [3.50–6.00]	3.00 [2.00–5.00]	< 0.001
Years	5.00 [3.00–8.50]	5.00 [2.00–7.00]	0.577
Performance			
Effort-adjusted speed (km-effort/h)	8.14 [7.63–9.82]	3.68 [3.56–3.86]	< 0.001
Activity duration (h)	7.33 [5.50–8.75]	7.50 [7.00–10.00]	0.003
RPE	8.00 [6.00–9.00]	5.00 [3.00–6.00]	< 0.001
Pain			
Maximum pain (NRS)	6.00 [3.00–7.50]	3.00 [1.00–5.00]	< 0.001
Average pain (NRS)	3.00 [1.00–5.00]	2.00 [0.50–3.00]	< 0.001
PCS	4.50 [2.50–6.25]	2.50 [0.75–4.00]	< 0.001
Pain threshold (s)	20.00 [10.00–51.00]	/	/
Pain tolerance (s)	180.00 [180.00–180.00]	/	/

*Note:* Data are presented as frequencies and medians with interquartile ranges (25th–75th percentiles). Differences between trail runners and hikers were evaluated using the Chi-square test and the Mann–Whitney *U* test, as appropriate. A *p* value of < 0.05 was considered statistically significant.

Abbreviations: BMI, Body Mass Index; NRS, Numeric Rating Scale; PCS, Pain Composite Score; RPE, Rating of Perceived Exertion.

**Table 2:** Comparison of demographics, training habits, and performance metrics between male and female recreational endurance athlete**s**.

	Male (*N* = 61)	Female (*N* = 61)	*p* value
Demographics			
Age (years)	40.00 [36.25–48.25]	38.00 [34.00–45.75]	0.126
BMI (kg/m^2^)	24.04 [22.35–25.62]	21.36 [19.95–23.23]	0.001
Training			
Hours per week	6.50 [4.00–10.00]	4.50 [2.00–8.00]	0.016
Days per week	4.00 [3.00–5.75]	4.00 [2.75–5.00]	0.481
Years	5.00 [2.75–10.50]	4.00 [2.00–7.00]	0.360
Performance			
Effort-adjusted speed (km-effort/h)	6.44 [3.71–8.66]	3.83 [3.57–5.48]	0.011
Activity duration (hours)	7.63 [6.79–9.56]	7.25 [7.00–10.00]	0.973
RPE	6.00 [4.00–8.00]	5.00 [3.00–7.00]	0.164
PCS	3.00 [1.00–5.00]	2.50 [2.00–4.50]	0.853

*Note:* Data are presented as medians with interquartile ranges (25th–75th percentile). Differences between male and female participants were assessed using the Mann–Whitney *U* test. A *p* value of < 0.05 was considered statistically significant.

Abbreviations: BMI, Body Mass Index; PCS, Pain Composite Score; RPE, Rating of Perceived Exertion.

**Table 3:** Univariate analysis of predictors of exercise-induced pain intensity.

Predictors	B	S.E.	95% C.I.	Exp (B)	*p* value
Demographics					
Sex (male)	0.773	0.3534	0.080–1.466	2.1663	0.029
Sex (female)	0.996	0.2671	0.472–1.519	2.7074	< 0.001
Age (years)	0.022	0.0054	0.012–0.033	1.0222	< 0.001
BMI (kg/m^2^)	0.040	0.0092	0.021–0.058	1.0408	< 0.001
Training					
Hours per week	0.134	0.0453	0.045–0.223	1.1434	0.003
Days per week	0.208	0.0607	0.089–0.327	1.2312	0.001
Years	0.110	0.0381	0.035–0.185	1.1163	0.004
Performance					
Sport (trail runners)	0.639	0.2421	0.164–1.113	1.8946	0.008
Effort-adjusted speed (km-effort/h)	0.249	0.0592	0.133–0.365	1.2827	< 0.001
Activity duration (h)	0.096	0.0235	0.050–0.142	1.1008	< 0.001
RPE	0.200	0.0443	0.114–0.287	1.2214	< 0.001
Pain threshold and tolerance					
Pain threshold (s)	−0.001	0.0024	−0.005–0.005	1.0000	0.994
Pain tolerance (s)	−0.001	0.0032	−0.007–0.005	0.9990	0.755

*Note:* Univariate generalized linear models (Gamma distribution, log link function) with pain composite score as the dependent variable. Significant predictors (*p* < 0.05) were identified based on Wald Chi-square tests, with larger B coefficients indicating stronger associations with pain intensity.

Abbreviations: BMI, Body Mass Index; RPE, Rating of Perceived Exertion.

**Table 4:** Multiple regression analyses of sex, sport and performance metrics as predictors of exercise-induced pain intensity.

Predictors	B	S.E.	95% C.I.	Exp (B)	*p* value
Model 1: Sex, sport and rating of perceived exertion					
Sex (male)	−0.293	0.2387	−0.761–0.175	0.7460	0.220
Sport (trail runners)	0.259	0.2770	−0.284–0.802	1.2956	0.350
RPE	0.204	0.0569	0.093–0.316	1.2263	< 0.001
Model 2: Sex, sport, and effort-adjusted speed (km-effort/h)					
Sex (male)	−0.194	0.2472	−0.679–0.290	0.8237	0.432
Sport (trail runners)	0.743	0.2910	0.173–1.313	2.1022	0.011
Effort-adjusted speed (km-effort/h)	−0.005	0.0193	−0.043–0.032	0.9950	0.782
Model 3: Sex, sport, and activity duration (h)					
Sex (male)	−0.203	0.2464	−0.685–0.280	0.8163	0.411
Sport (trail runners)	0.717	0.2715	0.175–1.249	2.0483	0.008
Activity duration (h)	0.008	0.0596	−0.109–0.124	1.0080	0.899

*Note:* Generalized linear models (Gamma distribution, log link function) with pain composite score as the dependent variable. Significant predictors (*p* < 0.05) were identified based on Wald Chi-square tests, with B coefficients representing the strength and direction of association with pain intensity.

Abbreviation: RPE, Rating of Perceived Exertion.

**Table 5:** Sex-specific interactions with pain threshold and tolerance in exercise-induced pain intensity.

Predictors	B	S.E.	95% C.I.	Exp (B)	*p* value
Model 1: Sex and pain threshold					
Sex (male)	−0.184	0.3961	−0.960–0.593	0.8319	0.643
Pain threshold (seconds)	−0.001	0.0033	−0.007–0.006	0.9990	0.788
Male × pain threshold	0.001	0.0052	−0.009–0.012	1.0010	0.785
Model 2: Sex and pain tolerance					
Sex (male)	7.621	18.7051	−29.040–44.282	2047.05	0.684
Pain tolerance (seconds)	−0.004	0.0063	−0.016–0.009	0.9962	0.571
Male × pain tolerance	−0.042	0.1040	−0.246–0.162	0.9594	0.687

*Note:* Generalized linear models (Gamma distribution, log link function) with the pain composite score as the dependent variable. Significant predictors (*p* < 0.05) were identified based on Wald Chi-square tests. B coefficients represent the strength and direction of the association with pain intensity.

## Data Availability

The data that support the findings of this study are available upon request from the corresponding author. The data are not publicly available due to privacy or ethical restrictions.
